# Top-down effects on translucency perception in relation to shape cues

**DOI:** 10.1371/journal.pone.0314439

**Published:** 2025-02-18

**Authors:** Takehiro Nagai, Hiroaki Kiyokawa, Juno Kim

**Affiliations:** 1 School of Engineering, Institute of Science Tokyo, Yokohama, Japan; 2 Graduate School of Science and Engineering, Saitama University, Saitama, Japan; 3 School of Optometry and Vision Science, University of New South Wales, Sydney, Australia; Shibaura Institute of Technology, JAPAN

## Abstract

It is well established that object shape perception significantly influences the perception of translucency. However, how object shape cues such as motion and binocular disparity affect the perception of translucency in rich environments, like virtual reality or real visual environments, remains unclear. This study aims to psychophysically measure the extent to which multiple object shape cues influence the perception of translucency. Additionally, we examined whether top-down factors, such as changes in cognitive attitude caused by the sequence of experiments, affect translucency perception. The results revealed that while motion and binocular disparity enhance translucency perception, this effect is confined to situations where shape cues are poor. Moreover, the effect became particularly pronounced when the experiments began with weak specular reflection stimuli, followed by the experiments using stimuli with specular reflection. In the case of translucent objects without specular reflection, strong shape information cannot be derived solely from shading patterns. These findings thus suggest that top-down factors related to shape modulate the influence of shape cues on translucency perception.

## Introduction

When humans observe objects, they form various impressions about the material properties. This process is referred to as “material perception” in this study. Unlike simple visual attributes such as shape and color, material perception involves the complex interactions between an object’s material, shape, and lighting, which generates diverse patterns in the retinal image [1]. Therefore, as with surface color perception, it is impossible to uniquely determine a material’s properties from the retinal image alone. As a result, research on material perception has primarily focused on identifying the heuristics the visual system employs to interpret material properties from the retinal image [2,3].

The properties of glossiness, a representative material feature, have been the subject of extensive research, particularly since the 2000s. Early studies identified a relationship between two-dimensional image features and glossiness perception [4–6], suggesting that the heuristics employed by the visual system might be relatively simple. However, later studies revealed that glossiness perception results from the interaction of surface image patterns with various factors specific to specular reflection, such as motion flow, binocular disparity, and other shape information [7–9]. These findings indicate that glossiness perception arises from a complex combination of multiple factors. Such multifactorial influences likely apply to other material features as well.

Research on the perception of translucency has been gradually increasing. While glossiness primarily reflects the surface’s reflective properties, translucency pertains to material perception associated with the light-transmitting characteristics of the material. Similarly, transparency, another perception related to light transmission, has been extensively studied, particularly in relation to object-background interactions, as exemplified by Metelli’s law [10]. In contrast, translucency perception emerges from the retinal image created by complex interactions between lighting, the object’s optical properties, and its shape. Consequently, much like glossiness, several studies have shown that complex spatial patterns of luminance and chromaticity serve as cues for translucency [11–14]. This can be attributed to the unique patterns caused by subsurface light scattering in translucent objects, which differ from those in opaque objects.

A key feature of translucency is its strong dependence on object shape. This is because translucency involves the perception of light transmission and scattering, whose appearance of object surfaces is directly influenced by the object’s three-dimensional shape. For example, when an object has both thin and thick parts, the direction of the light source has a greater impact on the perception of translucency [15]. Moreover, it has been shown that the combination of shape perception and luminance patterns is a powerful cue for evoking the perception of translucency [16]. While motion and binocular disparity are commonly used as cues for shape perception, the presence of specular highlights also provides strong shape information [17,18]. This effect is thought to be particularly strong in translucent objects, as subsurface scattering reduces the contrast of luminance patterns on object surfaces [19], making it difficult to derive shape information from shading alone [20]. Indeed, when specular highlights are present, shape perception of translucent objects improves, and perceptual translucency is enhanced as well [21].

How does visual information related to shape contribute to the perception of translucency? For instance, in studies demonstrating the influence of shape on translucency, it was shown that even when object stimuli possess identical luminance patterns, significantly altering the perceived shape through binocular disparity can affect translucency perception [16]. This strongly suggests that shape perception plays a crucial role in translucency. However, in more typical situations where various cues, such as object shape contours, are available, the ambiguity of shape perception in translucent objects is relatively low. A straightforward hypothesis is that the perceived shape, resulting from the integration of all available cues, combines with luminance patterns to produce the perception of translucency. In such cases, even a single effective cue for shape perception may suffice to significantly impact translucency perception. Furthermore, if shape recognition is indeed critical, top-down factors related to shape recognition could potentially alter the perception of translucency, even when the visual stimuli remain identical. As mentioned above, the surface luminance contrast in translucent objects is inherently reduced, which diminishes the availability of visual cues critical [19]. In such situations, prior knowledge about objects and expectations formed through the presentation history of visual stimuli may significantly influence shape-related cognition. These top-down influences could modify shape perception and, consequently, affect the perception of translucency. If this is the case, offering comprehensive prior knowledge or incorporating multisensory information about the object may be crucial for accurately reproducing translucency perception. These insights are likely important for applications such as accurately replicating translucency perception in Virtual Reality (VR) environments.

This study aims to elucidate how multiple object shape cues interact and contribute to the perception of translucency. We focus on factors relevant to VR—specifically, object motion and binocular disparity—and investigate their complementary effects on translucency by systematically controlling their intensity or presence in psychophysical experiments. While previous research has demonstrated the contribution of object shape cues to translucency perception, these investigations typically involved drastic manipulations in shape perception using cues like motion [16]. In contrast, this study seeks to determine the relative influence of these cues where they are naturally added. The second aim of this study was to investigate whether shape recognition provides a top-down influence on the effects of binocular disparity and motion on translucency perception. To address this, observers were divided into two groups with different stimulus presentation sequences, and the relationship between shape cues and translucency was compared between the groups. In one group, the experiment started under conditions with strong specular highlights, where shape cues were abundant. In the other group, the experiment began under conditions with weak specular highlights, where shape information remained insufficient despite the presence of object motion and binocular disparity. Since both groups observed the same experimental stimuli, any differences in their translucency evaluations should be attributed to differences in shape recognition arising from the order of presentation. Although such top-down factors can often occur in daily life and psychophysical experiments due to experimental order or prior knowledge, they have not been directly examined in previous research. Thus, if top-down factors are found to significantly affect translucency perception, this would highlight critical considerations for future studies on translucency perception (and material perception in general, possibly).

## Methods

### Observers

Ten undergraduate and graduate students from the Institute of Science Tokyo (two of whom were women aged 22–24) participated in the experiment. All observers had normal or corrected-to-normal vision and were unaware of the experiment’s purpose. As detailed later, observers were randomly assigned to two groups of five, with each group following a different experimental order. All observers were Japanese, a deliberate choice to minimize the potential influence of cultural differences on the comparison of results between the two groups. The experiment was conducted in accordance with the Declaration of Helsinki and approved by the Ethical Review Committee of Institute of Science Tokyo. The observers were recruited between May 7, 2024, and June 11, 2024, and all experiments were completed within this period.

### Apparatus

**Fig 1:**
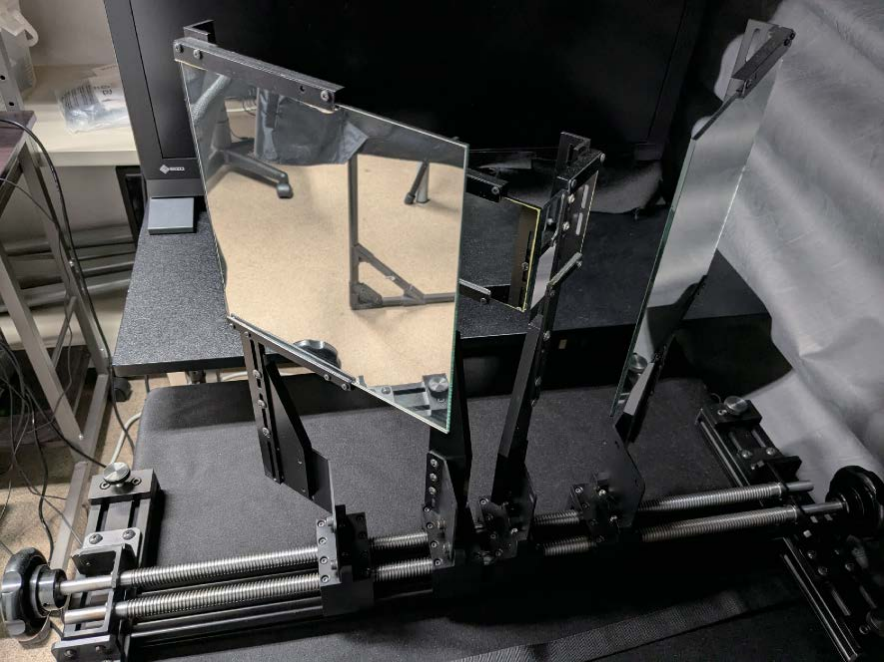
Mirror stereoscope.

The experiment was conducted in a simple darkroom equipped with blackout curtains. An LCD display (CG279X, EIZO, Japan) was used, featuring a spatial resolution of 2560 ×  1440 pixels and a frame rate of 60 Hz. The display’s color gamut was set to sRGB with a white point at D65, a maximum brightness of 120 cd/m², and a gamma of 2.2, calibrated using the display’s self-calibration function. These settings were verified using a spectroradiometer (Specbos 1211-2, JETI, Germany). A custom-made mirror stereoscope shown in Fig 1 was positioned in front of the display, allowing the stimuli on the left and right halves of the screen to be directed to the left and right eyes, respectively. This stereoscope comprised four mirrors positioned at ± 45 degrees relative to the display surface, with two mirrors designated for each eye. The inner mirrors, measuring 76 mm ×  76 mm, were silver-coated mirrors (Edmund Optics, USA), while the outer mirrors, measuring 200 mm ×  200 mm, were enhanced aluminum-coated mirrors from the same manufacturer. The inner pair of mirrors were placed adjacent to each other, and the two mirrors assigned to each eye were separated by 13 cm from the corresponding inner mirror along the optical path for each eye. The optical viewing distance to the display through the stereoscope was 50.6 cm. Observers’ heads were stabilized with a chin rest for consistent fixation.

The entire experimental procedure was controlled by a computer (GV301Q, equipped with an AMD Ryzen™ 7 5800HS processor and an NVIDIA® GeForce® GTX 1650 GPU, ASUS, Taiwan). The experiment was managed using a custom program developed with the Coder component of PsychoPy [22]. Observers provided their responses using a trackball.

### Stimuli

**Fig 2:**
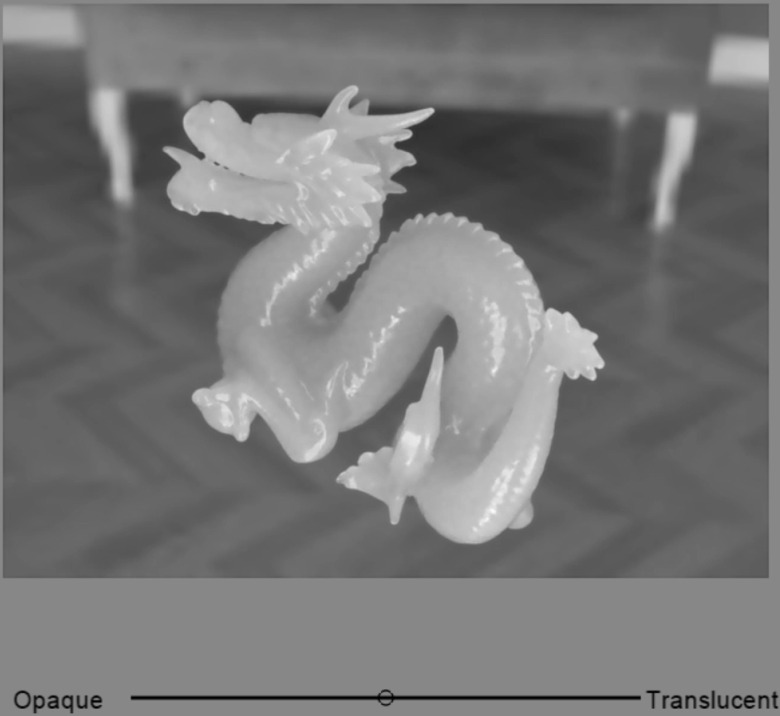
Example of a stimulus. This figure shows the stimulus presented to the left eye only. The Visual Analog Scale (VAS) scale bar and cursor at the bottom of the display were presented in both the left and right eye stimuli, with zero binocular disparity.

Fig 2 presents an example of the stimulus. The stimuli consisted of either still images or videos on based computer graphics (CG). Below the stimulus, a black bar (R, G, B =  0, 0, 0) and a small circular cursor were displayed, allowing observers to respond using the Visual Analog Scale (VAS). The background was a uniform gray with a luminance of 32.7 cd/m² and a D65 chromaticity.

### Computer graphics rendering

The stimuli for each object were created from 800 ×  600 pixel CG images rendered using Mitsuba 3 [23]. Since this study does not focus on the effect of color on translucency perception (e.g., [24]), the CG images were rendered in RGB rather than spectrum-based. An environment emitter was used for lighting, and the environment map “Brown Photostudio 06,” obtained from Poly Haven (https://polyhaven.com/a/brown_photostudio_06), was employed. To create video stimuli depicting object motion, 91 images were rendered by rotating the object from -45 degrees to + 45 degrees in 1-degree increments. For stereoscopic viewing, these 91 images were rendered from two separate positions, assuming an interocular distance of 6.2 cm (unit distance 62), with the camera aimed at the object placed at the spatial origin. The distance from the camera to the object’s center was set at a unit distance of 506. Specifically, the cameras were positioned at (X, Y, Z) =  (±31, 385, 330) (all in unit distance).

**Fig 3:**
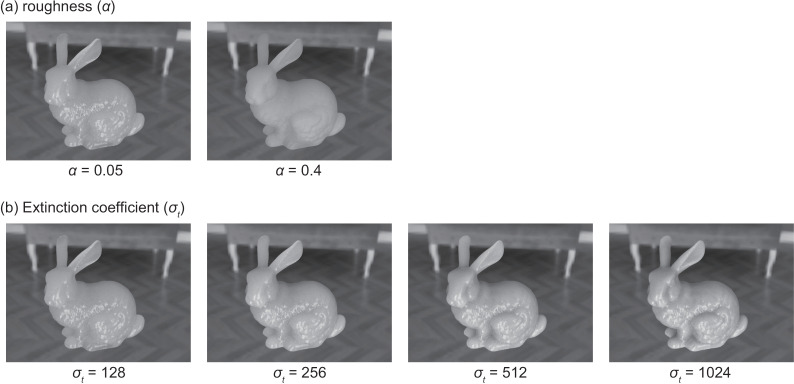
Variations in stimuli based on optical properties. (a) Changes in stimuli due to surface roughness (alpha). The left image shows α=0.05 while the right image shows *α* = 0.4. (b) Changes in stimuli based on the combination of single scattering albedo and the extinction coefficient. From left to right, the extinction coefficient $\sigma_{t}$ increases.

The Bidirectional Scattering Distribution Function (BSDF) for all objects was set to *roughdielectric*, a built-in BSDF model in Mitsuba. Participating media were configured inside the objects to represent light transmission and scattering. The surface roughness parameter *α* was set to 0.05 and 0.4, corresponding to strong and weak specular highlights, respectively. For convenience, we will refer to the condition with strong highlights as the “Specular” condition and the condition with weak highlights as the “Diffuse” condition. Fig 3a shows examples of images rendered with these two different *α* values. The parameters for the participating media were set using a combination of the single scattering albedo (*α*) and the extinction coefficient $\sigma_{t}$ Specifically, the RGB values for *α* were kept constant, and the combinations of (*α*, $\sigma_{t}$ were (0.966, 128), (0.985, 256), (0.995, 512), and (1.000, 1024). These values were chosen arbitrarily by the authors based on observations of the stimuli to induce variations in translucency. Fig 3b presents examples of images generated with these four combinations of *α* and $\sigma_{t}$

**Fig 4:**
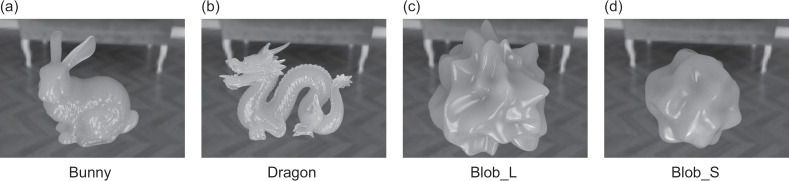
Examples of CG images generated from the four object shapes. (a) Bunny, (b) Dragon, (c) Blob_L, and (d) Blob_S.

There were four types of object shapes. Two of them, the Stanford Bunny and Dragon, were obtained from the Stanford 3D Scanning Repository (https://graphics.stanford.edu/data/3Dscanrep/). The other two shapes were blob forms created in Blender 4.1 by applying a cloud texture modifier to a UV sphere. The shape with larger surface bumps is referred to as Blob_L, while the one with smaller bumps is called Blob_S. The size of each object was adjusted to approximately 25 units in depth, width, and height. Fig 4 shows examples of CG images generated from these four shapes. Consequently, the images consisted of combinations of two surface roughness levels, four types of participating media, and four shapes.

No tone mapping was applied to the generated images. The CG images were saved as PNG files in sRGB format with a gamma of 2.2 and a D65 white point. In some pixels, the RGB values exceeded the maximum (255 in 8-bit format), and in such cases, the RGB intensity was clipped to 255. These clipped pixels were primarily located in the specular highlight areas.

### Creation of stimulus videos

Next, experimental stimuli were created using CG images. As a preparatory step, since the environment map contained colors, the rendered CG images included chromaticity information. To remove color cues, the chromaticity of all pixels was adjusted to D65 with CIE1931 (x, y) =  (0.313, 0.329), while maintaining their luminance. From this point, the experimental stimuli included two variations each for contour (Full and Masked), motion (Dynamic and Static), and binocular disparity (with and without disparity, called wDisparity and woDisparity, respectively). The Full and Masked conditions were introduced to manipulate 3D shape cues, as the presence or absence of object contours significantly affects the perception of 3D shape details [20]. The procedure for creating the Full condition stimuli was as follows. First, the images were resized to 600 ×  450 pixels using bicubic interpolation. Then, for each eye, a series of images from -45° to + 45° was compiled into a video oscillating between these angles at 30 frames per second, saved in H264 format as an mp4 video. In the Dynamic condition, the phase of the oscillation was synchronized between both eyes and randomly determined for each trial. In the Static condition, a single random frame was extracted from the video and presented as a still image in each trial. In the woDisparity condition, the stimulus intended for the left eye was presented to both eyes, creating a zero binocular disparity condition. The visual angle of the stimulus, including the background (part of the environment map), was 15.8° ×  11.9°, with the object itself measuring approximately 8.6° to 11.5° in width. In this study, the environment map behind the object in the rendered images was retained as the background. This decision aligns with one of the study’s objectives: to investigate how motion and binocular disparity contribute to the perception of translucency in naturalistic visual settings, such as those in VR.

The method for creating the stimuli in the Masked condition is described here. The procedure was nearly identical to that of the Full condition, with only a minor difference in the preprocessing step. First, the stimulus size was kept at the original 800 ×  600 resolution, and the chromaticity of all pixels was converted to D65. Then, all pixels outside a circle with a diameter of 240 pixels (visual angle of 6.4°), centered at (x, y) =  (400, 400), were replaced with a gray background. This effectively clipped most of the object image, leaving only the central region visible and making it difficult to use contour information for shape recognition. For most objects, the circular clipped area contained only the object itself; however, in the case of the Dragon, parts of the background from the environment map were occasionally visible, depending on the object’s angle. The circular clipping was positioned slightly lower than the center to maximize the area occupied by the object within the circle.

**Fig 5:**
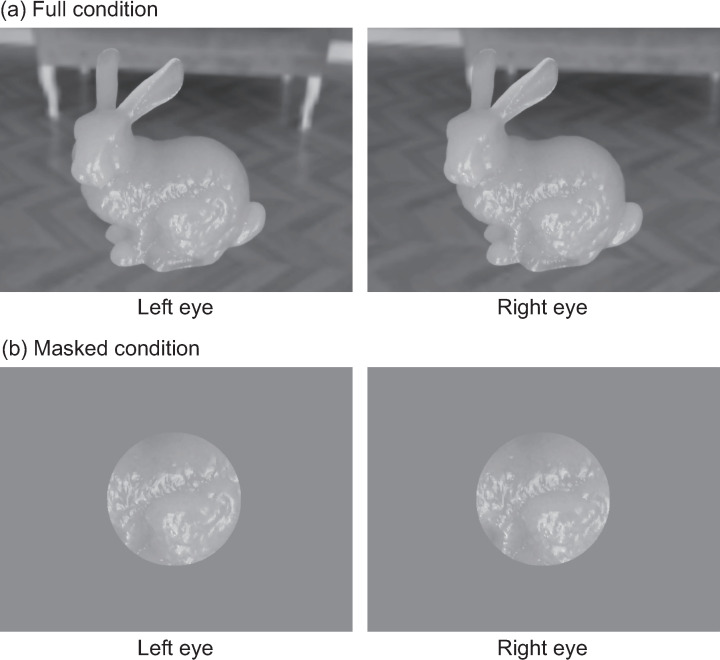
Examples of experimental stimuli. (a) Full and (b) Masked conditions. The left and right images correspond to the left and right eye stimuli, respectively. The example shown in this figure is of the Bunny under the conditions of *α* = 0.05 and $\sigma_{t}$= 128.

Fig 5 presents examples of experimental stimuli for both the Full and Masked conditions. Additionally, S1 Movie and S2 Movie in Supplementary Materials show the videos for the Full and Masked conditions, respectively. Supplementary Materials also include S1 and S2 Figs, which display the left-eye stimuli for all conditions. Furthermore, to show the image characteristics of the stimuli, S3 Fig presents the luminance histograms of the Bunny images as examples, while S4 Fig illustrates their low-order moment statistics, including the mean, RMS contrast, and skewness.

### Procedure

The experiment involved rating perceived translucency using a Visual Analog Scale (VAS). In each trial, a video was presented in the Dynamic condition and a still image in the Static condition, along with a VAS response bar. Observers used a trackball to move the response cursor left or right to adjust the scale according to their perceived level of translucency, and they clicked to submit their response once satisfied. The next stimulus appeared one second later. The stimuli remained visible until the observer completed their response, with no time limit imposed, as observers generally require more time to respond in rating experiments compared to, for instance, a two-alternative forced-choice (2AFC) task.

The four combinations of Specular/Diffuse and Full/Masked conditions were measured in separate sessions with two sessions per condition, totaling eight experimental sessions. Before beginning the trials in each session, observers were allowed to examine the stimulus set as much as they wanted, enabling them to establish criteria for judging translucency. Observers were instructed to use the full range of the VAS scale (from maximum to minimum) within each session and to maintain distinct judgment criteria for each session, without applying the same standards across sessions. Once observers had finalized their criteria, they informed the experimenter, and the trials began. Each session included 64 different stimuli based on four values of $\sigma_{t}$ four object shapes, two motion conditions (Dynamic vs. Static), and two binocular disparity conditions (wDisparity and woDisparity). Each stimulus was presented twice, resulting in 128 trials per session. The order of 128 trials was randomized in each session.

Additionally, observers were divided into two groups of five, each following a different session order. The first group began with the Specular condition. Specifically, they completed 2 sessions of Masked-Specular, followed by 2 sessions of Full-Specular, 2 sessions of Masked-Diffuse, and 2 sessions of Full-Diffuse. Starting with the Specular condition allowed this group to obtain object shape information early, as the Specular condition provides clearer shape cues. The second group followed the reverse order of Specular and Diffuse conditions; they completed 2 sessions of Masked-Diffuse, 2 sessions of Full-Diffuse, 2 sessions of Masked-Specular, and 2 sessions of Full-Specular. In this case, particularly during the Diffuse condition, even with motion or binocular disparity, the shape cues were insufficient, leading to a reduction in perceived surface details compared to reality [20]. The first group is referred to as the “specular-first group” and the second as the “diffuse-first group.”

### Analysis

For each observer’s results, the four session types (Full-Specular, Masked-Specular, Full-Diffuse, and Masked-Diffuse) were analyzed separately due to differences in judgment criteria across sessions. First, for each session type, the data for each observer were standardized to have a mean of 0 and a variance of 1, making the rating scales the same across observers. Before this standardization, we verified that response distributions were not excessively skewed or contained outliers. Afterward, the group average across observers was calculated. This result is referred to as the “normalized rating.” It is important to note that the normalized rating reflects only the relative translucency perception within each session and does not correspond to the absolute level of translucency across sessions. Therefore, comparisons of translucency can only be made between conditions, such as shape, $\sigma_{t}$ motion, and binocular disparity, within each session type, but not between glossiness conditions or between Full and Masked conditions. Consequently, no statistical tests were conducted across session types.

Statistical analysis was conducted using a non-parametric bootstrap method. The bootstrap procedure consisted of: (1) random sampling with replacement of observers within each group, and (2) random sampling of responses within each observer, to account for both between-observer and within-observer variance. The number of resampling iterations was set to 10,000. The significance level was *α* =  0.05, and unless otherwise specified, two-tailed tests were applied. In cases involving multiple comparisons, the Holm method was used to adjust the significance level, along with pairwise tests between conditions.

## Results

**Fig 6:**
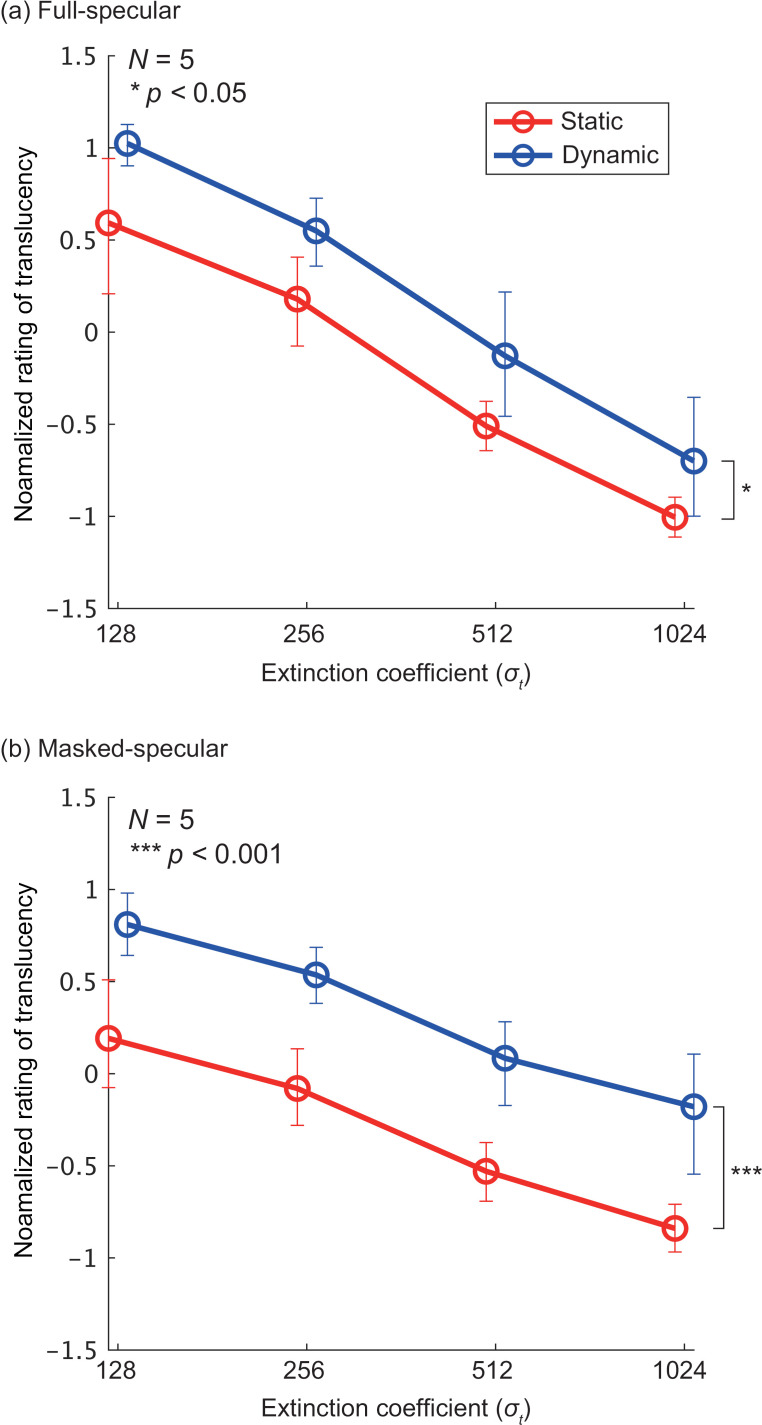
Normalized rating as a function of extinction coefficient $\sigma_{t}$ in the diffuse-first group. (a) Full-Specular condition and (b) Masked-Specular condition. The horizontal axis represents $\sigma_{t}$ and the vertical axis represents the normalized rating averaged across the shape and binocular disparity conditions. The colors in the graph distinguish between the Dynamic and Static conditions. Error bars represent the 95% confidence intervals obtained via bootstrapping. Note that the vertical axis scales differ between (a) and (b), so direct comparisons of translucency between the two cannot be made.

Fig 6 provides an example by showing the normalized ratings for the Full-Specular and Masked-Specular conditions in the diffuse-first group. It is important to note that, as with all normalized ratings in this study, observers applied different judgment criteria for translucency in the Full/Masked and Specular/Diffuse conditions. As a result, translucency magnitudes cannot be directly compared across these conditions, and the vertical axis scales also differ. As expected, smaller values of $\sigma_{t}$ (i.e., less absorption and scattering) corresponded to higher perceived translucency. Linear regression was performed for each graph (all four lines), and a one-sided bootstrap test was conducted to assess the significance of the slope relative to zero (*p* <  0.001 for all conditions). In all conditions, the slopes were significantly negative. This suggests that observers were able to perceive the physical property represented by $\sigma_{t}$ as translucency. Additionally, the Dynamic condition showed higher translucency perception than the Static condition. Testing the difference in normalized ratings between Static and Dynamic, averaged over $\sigma_{t}$ revealed statistically significant differences in both the Full-Specular (*p* <  0.05) and Masked-Specular (*p* <  0.001) conditions. This finding indicates that object motion enhances the perception of translucency.

**Fig 7:**
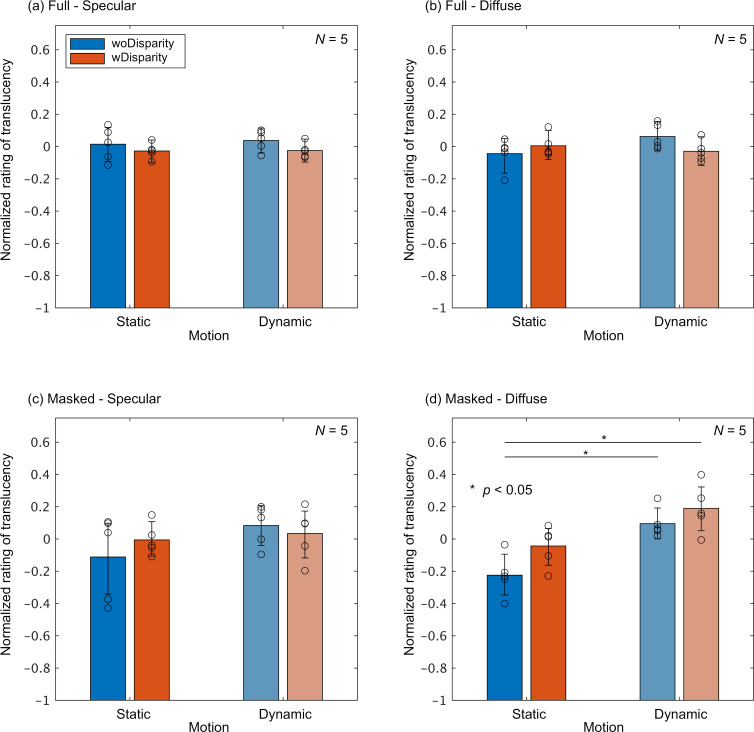
Normalized ratings averaged across shapes and$\sigma_{t}$for the specular-first group in the following conditions. (a) Full-Specular, (b) Masked-Specular, (c) Full-Diffuse, and (d) Masked-Diffuse. In each graph, the color of the bars represents the combination of motion and binocular disparity conditions. The vertical axis displays the normalized rating, with bar heights indicating the group mean and circular markers representing the results of individual observers. Error bars denote the 95% confidence intervals, which were estimated through bootstrapping. Statistically significant differences between conditions are indicated by asterisks.

Next, we compare the effects of motion and binocular disparity between the observer groups. Fig 7 shows the normalized ratings for the specular-first group, with the results averaged across the shapes and $\sigma_{t}$ This averaging process is consistent with the assessment of the respective main effects of motion and binocular disparity, confirming its methodological validity. In most panels, except for the Masked-Diffuse condition, the influence of disparity and motion appears minimal. A multiple comparison test using bootstrapping was conducted to evaluate statistical differences between the combinations of motion and disparity conditions in each panel. A statistically significant difference was observed only in the Masked-Diffuse condition, specifically between the Static-woDisparity condition and both Dynamic conditions.

**Fig 8:**
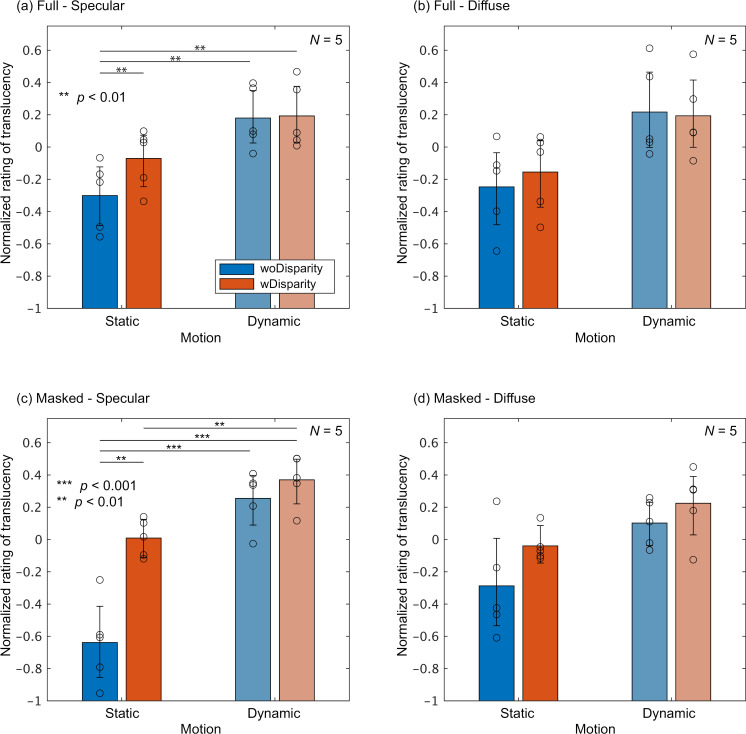
Normalized ratings averaged across shape and$\sigma_{t}$for the diffuse-first group. The format of the graphs is the same as in Fig 7.

Fig 8 presents the normalized ratings for the diffuse-first group. In this group, across all panels, the Dynamic condition consistently resulted in higher translucency perception than the Static condition, and wDisparity led to higher translucency than woDisparity. Similar to the specular-first group, statistical tests were conducted to evaluate the differences between motion and binocular disparity conditions. In the Full-Specular and Masked-Specular conditions, as indicated by the asterisks in Figs 8(a) and 8(c), many conditions exhibited statistically significant differences (all *p* <  0.01 or *p* <  0.001). Although no statistically significant differences were observed in the Full-Diffuse and Masked-Diffuse conditions, the Static-woDisparity condition produced the lowest normalized ratings in both cases.

**Fig 9:**
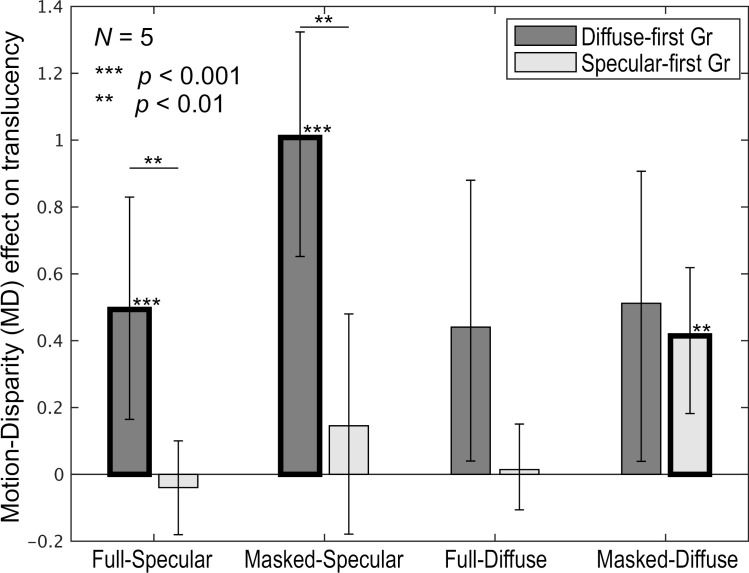
MD effect. The horizontal axis represents the combination of Full/Masked and Specular/Diffuse conditions, while the vertical axis displays the MD effect. The bar colors correspond to the observer groups. Error bars denote the 95% confidence intervals, estimated via bootstrapping. Asterisks above the bars and bold outlines indicate that the MD effects for those conditions are statistically significantly greater than zero. Asterisks spanning both the Diffuse-first and Specular-first groups indicate a significant difference in MD effect between the two groups. The results are averaged across shape and $\sigma_{t}$.

As shown, the effects of disparity and motion appear to differ between the groups. To quantify and compare these effects, we first calculated the difference in normalized ratings between the Dynamic-wDisparity and Static-woDisparity conditions, averaged across shape and $\sigma_{t}$ This difference captures the combined impact of motion and binocular disparity on translucency perception, which we refer to as the “MD (Motion & Disparity) effect.” The MD effect reflects the summation of the main effects of motion and binocular disparity. Fig 9 shows the MD effects for both observer groups across all conditions. For the specular-first group, the MD effect was close to zero in all conditions, and no statistically significant difference from zero was found using a one-sided bootstrap test, except in the Masked-Diffuse condition. In contrast, the diffuse-first group showed positive MD effects in almost all conditions, with statistically significant positive effects in the Full-Specular and Masked-Specular conditions. When comparing the groups, the MD effect appeared larger for the diffuse-first group than for the specular-first group in all conditions. Statistically significant differences between the groups were found in the Full-Specular and Masked-Specular conditions. These results suggest that the effects of motion and binocular disparity are stronger in the diffuse-first group, particularly when the object has strong specular reflection components.

**Fig 10:**
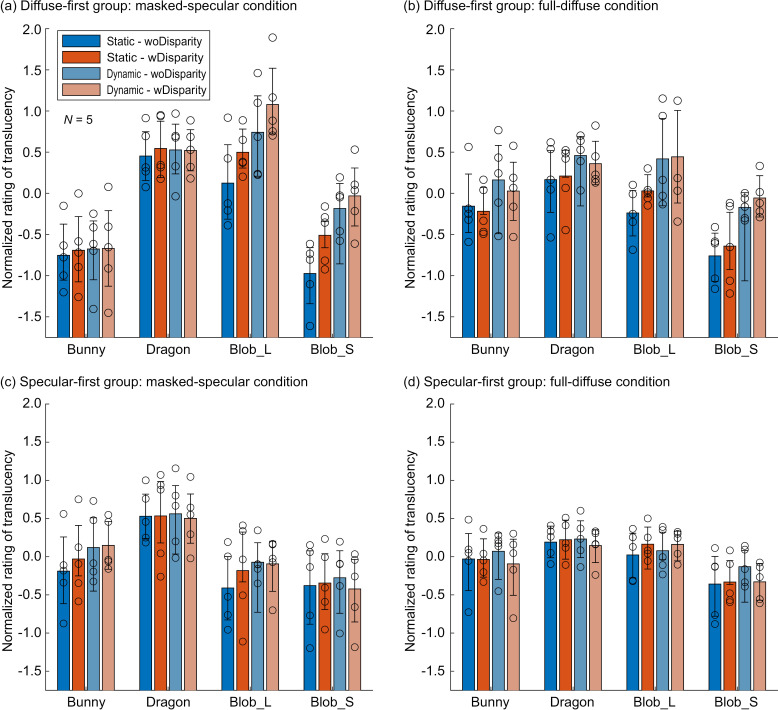
Normalized ratings for each object shape. (a) Masked-Specular condition for the diffuse-first group, (b) Full-Diffuse condition for the diffuse-first group, (c) Masked-Specular condition for the specular-first group, and (d) Full-Diffuse condition for the specular-first group. In each panel, the horizontal axis represents the object shape, and the vertical axis shows the normalized rating. The color of the bars corresponds to the motion and disparity conditions. Bar heights represent the group average, while circular markers indicate individual observer results. In all panels, the results are averaged across the Extinction coefficient. Error bars represent the 95% confidence intervals estimated via bootstrapping.

Lastly, to gain insights into the mechanisms underlying the effects of motion and binocular disparity, we compared these effects across different object shapes. Fig 10 presents the normalized ratings for each object shape. As examples, the Masked-Specular condition for the diffuse-first group and the specular-first group are shown in Figs 10a and 10c, and the Full-Diffuse condition results are shown in Figs 10b and 10d, respectively. These two conditions were selected because they represent the largest and smallest MD effects in the diffuse-first group, as indicated in Fig 9.

Several key points can be drawn from this figure. First, across all graphs, the normalized ratings for the Bunny and Dragon shapes show minimal differences between the motion and disparity conditions. In contrast, for the diffuse-first group, the normalized ratings for the two Blob shapes increase with the addition of each cue (motion and binocular disparity). When the MD effect (the difference in normalized ratings between Static-woDisparity and Dynamic-wDisparity) was tested for each shape, the Full-Diffuse condition, which had a small overall MD effect, showed statistically significant differences only for the two Blob shapes (*p* <  0.05 for Blob_L and *p* <  0.001 for Blob_S). These findings suggest that the influence of motion and binocular disparity on translucency perception is highly dependent on object shape.

Next, for Blob_L and Blob_S, the changes in normalized ratings due to motion and binocular disparity were nearly absent in the specular-first group but were clearly evident in the diffuse-first group. In the diffuse-first group (Figs 10a and 10b), the MD effect was statistically significantly greater than zero for both Blob_L and Blob_S (*p* <  0.05 for Blob_L and *p* < 0.001 for Blob_S under the Full-Diffuse condition, as noted earlier, and *p* <  0.001 for both Blob shapes under the Masked-Specular condition). Conversely, in the specular-first group (Figs 10c and 10d), the MD effect for the same conditions was not statistically significant for both Blob shapes and both conditions. These results suggest that the diminishing effects of motion and binocular disparity due to the experimental sequence (i.e., the difference between the observer groups) are evident even in shapes and conditions where these cues typically have a substantial impact on translucency perception.

## Discussion

In this study, we investigated how motion and disparity cues that provide 3D shape information contribute to translucency perception on stimuli where they were naturally added and whether these contributions can be modulated by top-down factors, such as cognitive expectations and prior knowledge induced by the experimental sequence. The experimental conditions included two motion types (Static and Dynamic) and the presence or absence of binocular disparity, both of which influence shape perception. To investigate the role of top-down factors, we focused on specular reflection components, which strongly provide shape information. We then divided observers into two groups: the Specular-first group, where the experiment started with Specular stimuli that facilitated shape recognition, and the Diffuse-first group, where the experiment started with Diffuse stimuli that made shape recognition more difficult. By comparing these two groups, we evaluated the impact of the experimental sequence on translucency perception. In this section, we first present the overall trends in how motion and binocular disparity influence translucency perception, considering differences in the shape and Full/Masked conditions. We then focus on top-down effects by comparing the influence of motion and binocular disparity on translucency perception between the Specular-first and Diffuse-first groups.

### General effects of motion and binocular disparity

As shown by the MD effect in Fig 9, in many conditions, the addition of motion and binocular disparity information significantly enhanced translucency perception. This finding aligns with previous research [16], which indicates that motion and binocular disparity can influence translucency perception by altering the perceived 3D shapes of objects. More specifically, motion and binocular disparity likely contributed to improved shape perception, which enhanced translucency perception. In objects with subsurface scattering, surface luminance contrast and the accompanying luminance gradients are reduced, diminishing the perception of surface details based on shading [20]. Motion and binocular disparity may compensate for this reduced shape perception, leading to more accurate shape recognition. Consequently, this could result in a more precise perception of translucency. These findings emphasize the importance of incorporating motion and binocular disparity cues to support accurate translucency perception.

However, it is important to note that the effects of binocular disparity and motion are highly condition-dependent. As shown in Fig 10, these effects were mainly observed in shapes like Blob_L and Blob_S, where the original shapes were difficult to estimate. In contrast, for the Dragon and Bunny, whose well-known shapes allow their complete 3D shapes to be easily inferred from contours alone, the effects of binocular disparity and motion were negligible. Additionally, as shown in Fig 9, the MD effect tended to be stronger in the Masked condition, where contour information could not be used for shape perception [20], compared to the Full condition. This trend was especially prominent in the Diffuse condition for the specular-first group and in the Specular condition for the diffuse-first group. Therefore, the effects of binocular disparity and motion are most pronounced when object shape is difficult to perceive or infer. On the other hand, when the shape is already familiar or can be sufficiently inferred from contours in static images, these effects are likely minimal. In environments rich in visual information, such as everyday scenes or VR environments, where multiple 3D shape cues like object contours and self-occlusion are available, the contribution of motion and binocular disparity to translucency perception is likely to be limited.

Our findings do not exclude the possibility of a mechanism for translucency perception based on two-dimensional image features, even though they demonstrate that shape cues have a significant influence on translucency perception. Although the relationship between two-dimensional image features and translucency perception has yet to reach a definitive conclusion, it has been investigated in numerous psychophysical studies [12,19,25–27]. Some studies have explored this relationship in the context of how two-dimensional image features influence translucency perception via three-dimensional shape information. For example, illusory translucency induced by specular highlights has been shown to correlate more strongly with luminance gradients related to shape than with shape perception itself [13]. Additionally, physical constraints between 3D shape and translucency suggest that these constraints may affect the perception of translucency from two-dimensional image features [21]. While our results indicate that 3D shape perception plays a significant role in translucency perception, we cannot rule out the possibility that translucency perception may arise directly from two-dimensional image features without reliance on 3D shape information. Further investigation is needed to clarify the specific processes involved in translucency perception from two-dimensional image features.

### Top-down factors: comparison of specular-first and diffuse-first groups

Another intriguing result of this study is the effects of binocular disparity and motion on translucency perception differed markedly depending on whether the experiment began with the Diffuse or Specular condition. Specifically, the diffuse-first group exhibited a much stronger enhancement in translucency perception due to motion and binocular disparity compared to the specular-first group. Since the stimuli were identical for both groups, with only the order of presentation varying, this indicates that certain top-down factors during stimulus observation influenced the impact of motion and binocular disparity. Notably, the difference in these effects between the groups was observed only for the Blob stimuli, implying that the top-down factor driven by the order of presentation is likely related to shape recognition.

The reason for the differences between the two observer groups cannot be concluded based on the current results alone. Our findings just indicate that initiating the experiment with the Diffuse condition plays a critical role in shaping the effects of motion and disparity on translucency perception. One plausible hypothesis for these observed top-down effects is that differences in shape perception induced by the experimental sequence modulated translucency perception. On the diffuse stimuli, the weak luminance shading should have resulted in a diminished perception of 3D surface bumps, even in the presence of motion and binocular disparity. In contrast, the specular stimuli provided abundant shape information via specular reflection components [17,18], allowing for more accurate shape perception (though binocular disparity of specular reflections may have distorted the perceived depth [28]). In the specular-first group, the early establishment of object shape recognition may have left little room for motion and binocular disparity to further enhance shape perception. To clarify this possibility, a reasonable next step would be to measure shape perception (e.g., the perceived magnitude of surface bumpiness) during the experiment as stated later, because this was not assessed in the current study. Alternatively, another hypothesis is that the priming effect heightened attention to specular highlights, which can serve as an important cue for shape perception. In the diffuse-first group, observers saw diffuse stimuli during the first half of the experiment and specular stimuli during the second half. This contrast may have drawn their attention to the novel and shape-rich [17,18] specular highlights in the latter half. Furthermore, the influence of shape cues, such as motion, tends to be stronger in the presence of specular highlights [29]. Consequently, in the diffuse-first group, the enhanced impact of motion and binocular disparity on shape recognition might have led to a stronger contribution to translucency perception. To investigate this possibility, future research could adopt a strategy involving the simultaneous execution of highlight-related tasks and translucency perception assessments. This approach could compel observers to focus on specular highlights, enabling an examination of their influence on shape and translucency perception. Additionally, tracking eye movements during the experiment and analyzing their correspondence with translucency perception could provide further insights into the role of cognitive factors.

Our results demonstrate only a part of potential top-down effects on translucency perception. In this experiment, we investigated order effects exclusively between the Specular-first and Diffuse-first groups, as this study was designed to focus on specular reflection components, which strongly influence shape perception. Similarly, it is highly likely that order effects also exist between the Full and Masked conditions, because the impacts of motion and binocular disparity were more pronounced in the Masked condition. However, because the Masked condition was always conducted before the Full condition, we were unable to evaluate the order effects between them. Investigating these order effects might reveal, for instance, that starting with the Masked condition could significantly hinder shape recognition, thereby amplifying the effects of motion and binocular disparity. Furthermore, dividing observers into groups based on whether or not they had prior knowledge of the object shapes could also uncover differences in how the knowledge influences translucency perception. Incorporating such experimental designs would provide deeper insights into whether the mechanisms of top-down influences on translucency perception are genuinely related to shape recognition.

Nevertheless, the findings of this study provide a strong example of the influence of higher-level recognition in material perception research. Much of the prior work on glossiness and translucency has focused on feedforward processing. For example, a previous study [30] demonstrated that material perception changes significantly based on the recognition of viewing distance, highlighting how feedback from higher-level recognition can influence material perception. Similarly, combining visual and auditory stimuli can dramatically alter material perception [31], and this multimodal interaction might also involve higher-level recognition. Additionally, in painting, expectations—such as the notion that “the Caribbean Sea appears more transparent”—have been suggested to affect translucency perception [32]. As noted above, although the order effects observed in our results are likely associated with shape, their precise origin remains inconclusive. However, they can be understood within a similar feedback framework, which may be flexible enough to change simply based on stimulus order. Therefore, even when focusing on bottom-up processing in material perception research, it is crucial to carefully control the top-down effects of recognition to avoid unexpected results.

### Limitations of this study and directions for future research

This study has several limitations that hinder the ability to draw definitive conclusions. First, the number of observers was limited. The study involved only 10 observers, divided into two groups of five, which restricts the generalizability of the findings. Increasing the number of observers in future studies would enhance statistical robustness and reliability. Second, the diversity of stimulus parameters was insufficient. This study revealed that the effects of motion and binocular disparity strongly interact with object shapes. Expanding the variety of object shapes in future research would enable a more detailed investigation of how motion and binocular disparity relate to different object shapes. Lastly, only achromatic stimuli were used in this study, despite previous research showing that color significantly contributes to translucency perception [12,33,34]. While the mechanisms underlying the influence of color on translucency remain unclear, one hypothesis suggests that the spatial distribution of saturation, resulting from repeated subsurface scattering, may play a role [12]. When rich 3D shape cues are available in colored stimuli, it may be easier to capture the correspondence between 3D spatial positions and color saturation, potentially enhancing translucency perception. Incorporating experiments with colored stimuli in future studies could yield new insights into top-down effects on translucency perception.

Finally, it is important to emphasize that this study did not provide direct evidence regarding the top-down information that influenced translucency perception through order effects. First, it is necessary to investigate whether the differences in translucency perception caused by presentation order are related to shape recognition. One approach would be to ask observers not only about translucency but also about their perceived shape (particularly surface bumps and indentations). Since perceived shapes may differ depending on the stimulus presentation order and between observers, this would allow for an exploration of the correlation between shape recognition and translucency perception. Measuring shape perception is frequently conducted in studies of material perception [17,20,21,35]. Additionally, manipulating visual attention to shape cues would be crucial for understanding their influence on translucency perception. Moreover, given that translucency perception spans multiple material categories, such as soap and candles, it may be constructed from several perceptual dimensions. If this is the case, it is possible that order effects influenced material recognition first, which then impacted translucency perception. This possibility could also be explored experimentally.

## Conclusions

This study aimed to clarify the impact of multiple shape-related cues, such as motion and binocular disparity, on translucency perception. Additionally, we investigated whether differences in shape recognition and cognitive attitude, influenced by the experimental order, affect translucency perception through top-down processes. The results demonstrated that motion and binocular disparity do enhance translucency perception, but their effect is limited to situations where shape-related cues are insufficient. Furthermore, this effect was notably limited in observers who initially experienced stimuli with strong specular reflection, while it was significantly stronger in those who first encountered stimuli with weak specular reflection. Given that specular reflection provides stronger shape cues than shading in translucent objects, these findings suggest that when shape recognition is incomplete due to prolonged exposure to weak specular reflection, top-down influences can increase the importance of shape cues in subsequent translucency judgments.

## Supporting information

S1 Movie Video for the Full condition. (MP4)

S2 Movie Video for masked condition. (MP4)

S1 Fig Images under Specular condition. (PDF)

S2 Fig Images under Diffuse condition. (PDF)

S3 Fig Luminance histogram of rendered images. (PDF)

S4 Fig Basic luminance statistics of rendered images. (PDF)

S1 File Inclusivity in global research questionnaire. (PDF)

## References

[1] Thompson W, Fleming R, Creem-Regehr S, Stefanucci JK. Visual perception from a computer graphics perspective. A K Peters/CRC Press; 2011.

[2] Nishida S. Image statistics for material perception. Curr Opin Behav Sci. 2019;30:94–9.

[3] Fleming RW. Visual perception of materials and their properties. Vision Res. 2014;94:62–75. doi: 10.1016/j.visres.2013.11.004 24291494

[4] Nishida S, Shinya M. Use of image-based information in judgments of surface-reflectance properties. J Opt Soc Am A Opt Image Sci Vis. 1998;15(12):2951–65. doi: 10.1364/josaa.15.002951 9857525

[5] Motoyoshi I, Nishida S, Sharan L, Adelson EH. Image statistics and the perception of surface qualities. Nature. 2007;447(7141):206–9. doi: 10.1038/nature05724 17443193

[6] Wiebel CB, Toscani M, Gegenfurtner KR. Statistical correlates of perceived gloss in natural images. Vision Res. 2015;115(Pt B):175–87. doi: 10.1016/j.visres.2015.04.010 25937518

[7] Wendt G, Faul F, Mausfeld R. Highlight disparity contributes to the authenticity and strength of perceived glossiness. J Vis. 2008;8(1):14.1–10. doi: 10.1167/8.1.14 18318617

[8] Sakano Y, Ando H. Effects of head motion and stereo viewing on perceived glossiness. J Vis. 2010;10(9):15. doi: 10.1167/10.9.15 21106677

[9] Marlow PJ, Todorović D, Anderson BL. Coupled computations of three-dimensional shape and material. Curr Biol. 2015;25(6):R221–2. doi: 10.1016/j.cub.2015.01.062 25784037

[10] Singh M, Anderson BL. Toward a perceptual theory of transparency. Psychol Rev. 2002;109(3):492–519. doi: 10.1037/0033-295x.109.3.492 12088242

[11] Gigilashvili D, Thomas J-B, Hardeberg JY, Pedersen M. Translucency perception: a review. J Vis. 2021;21(8):4. doi: 10.1167/jov.21.8.4 34342646 PMC8340651

[12] Fleming RW, Bülthoff HH. Low-level image cues in the perception of translucent materials. ACM Trans Appl Percept. 2005;2(3):346–82. doi: 10.1145/1077399.1077409

[13] Kiyokawa H, Nagai T, Yamauchi Y, Kim J. The perception of translucency from surface gloss. Vision Res. 2023;205:108140. doi: 10.1016/j.visres.2022.108140 36336645

[14] Gkioulekas I, Walter B, Adelson EH, Bala K, Zickler T. On the appearance of translucent edges. Proc IEEE Conf Comput Vis. Pattern Recognit. 2015:5528–36.

[15] Xiao B, Walter B, Gkioulekas I, Zickler T, Adelson E, Bala K. Looking against the light: how perception of translucency depends on lighting direction. J Vis. 2014;14(3):17. doi: 10.1167/14.3.17 24627457

[16] Marlow PJ, Kim J, Anderson BL. Perception and misperception of surface opacity. Proc Natl Acad Sci U S A. 2017;114(52):13840–5. doi: 10.1073/pnas.1711416115 29229812 PMC5748181

[17] Norman JF, Todd JT, Orban GA. Perception of three-dimensional shape from specular highlights, deformations of shading, and other types of visual information. Psychol Sci. 2004;15(8):565–70. doi: 10.1111/j.0956-7976.2004.00720.x 15271003

[18] Norman JF, Phillips F, Cheeseman JR, Thomason KE, Ronning C, Behari K, et al. Perceiving object shape from specular highlight deformation, boundary contour deformation, and active haptic manipulation. PLoS One. 2016;11(2):e0149058. doi: 10.1371/journal.pone.0149058 26863531 PMC4749382

[19] Motoyoshi I. Highlight-shading relationship as a cue for the perception of translucent and transparent materials. J Vis. 2010;10(9):6. doi: 10.1167/10.9.6 20884604

[20] Chowdhury NS, Marlow PJ, Kim J. Translucency and the perception of shape. J Vis. 2017;17(3):17. doi: 10.1167/17.3.17 28355629

[21] Marlow PJ, Anderson BL. The cospecification of the shape and material properties of light permeable materials. Proc Natl Acad Sci U S A. 2021;118(14). 10.1073/pnas.2024798118 PMC8040810 33811143

[22] Peirce J, Gray JR, Simpson S, MacAskill M, Höchenberger R, Sogo H, et al. PsychoPy2: experiments in behavior made easy. Behav Res Methods. 2019;51(1):195–203. doi: 10.3758/s13428-018-01193-y 30734206 PMC6420413

[23] Jakob W, Speierer S, Roussel N, Nimier-David M, Vicini D, Zeltner T, et al. Mitsuba 3 renderer (Version 3.0.1) [Computer software]; 2022.

[24] Chadwick AC, Cox G, Smithson HE, Kentridge RW. Beyond scattering and absorption: perceptual unmixing of translucent liquids. J Vis. 2018;18(11):18. doi: 10.1167/18.11.18 30372728 PMC6205562

[25] Nagai T, Ono Y, Tani Y, Koida K, Kitazaki M, Nakauchi S. Image regions contributing to perceptual translucency: A psychophysical reverse-correlation study. Iperception. 2013;4(6):407–28. doi: 10.1068/i0576 24349699 PMC3859557

[26] Liao C, Sawayama M, Xiao B. Unsupervised learning reveals interpretable latent representations for translucency perception. PLoS Comput Biol. 2023;19(2):e1010878. doi: 10.1371/journal.pcbi.1010878 36753520 PMC9942964

[27] Cai Y, Kiyokawa H, Nagai T, Haghzare L, Arnison M, Kim J. Effects of specular roughness on the perception of color and opacity. J Opt Soc Am A Opt Image Sci Vis. 2023;40(3):A220–9. doi: 10.1364/JOSAA.479972 37133045

[28] Muryy AA, Welchman AE, Blake A, Fleming RW. Specular reflections and the estimation of shape from binocular disparity. Proc Natl Acad Sci U S A. 2013;110(6):2413–8. doi: 10.1073/pnas.1212417110 23341602 PMC3568321

[29] Dövencioglu DN, Ben-Shahar O, Barla P, Doerschner K. Specular motion and 3D shape estimation. J Vis. 2017;17(6):3. doi: 10.1167/17.6.3 28586897

[30] Cheeseman JR, Fleming RW, Schmidt F. Scale ambiguities in material recognition. iScience. 2022;25(3):103970. doi: 10.1016/j.isci.2022.103970 35281732 PMC8914553

[31] Fujisaki W, Goda N, Motoyoshi I, Komatsu H, Nishida S. Audiovisual integration in the human perception of materials. J Vis. 2014;14(4):12. doi: 10.1167/14.4.12 24744448

[32] Wijntjes M, Spoiala C, De Ridder H. Thurstonian scaling and the perception of painterly translucency. Art Percept. 2020;1(aop):1–24.

[33] Liao C, Sawayama M, Xiao B. Crystal or jelly? Effect of color on the perception of translucent materials with photographs of real-world objects. J Vis. 2022;22(2):6. doi: 10.1167/jov.22.2.6 35138326 PMC8842421

[34] Chadwick AC, Cox G, Smithson HE, Kentridge RW. Beyond scattering and absorption: perceptual unmixing of translucent liquids. J Vis. 2018;18(11):18. 10.1167/18.11.18 PMC6205562 30372728

[35] Ohara M, Kim J, Koida K. The effect of material properties on the perceived shape of three-dimensional objects. i-Perception. 2020;11(6):2041669520982317. doi: 10.1177/2041669520982317 33489077 PMC7768321

