# Supplementary material for: Top-down effects on translucency perception in relation to shape cues
Source: PLoS One. 2025 Feb 18;20(2):e0314439. doi: 10.1371/journal.pone.0314439 (PMC11835294; doi:10.1371/journal.pone.0314439)
Supplement: S1 Fig — (PDF) [file pone.0314439.s003.pdf]

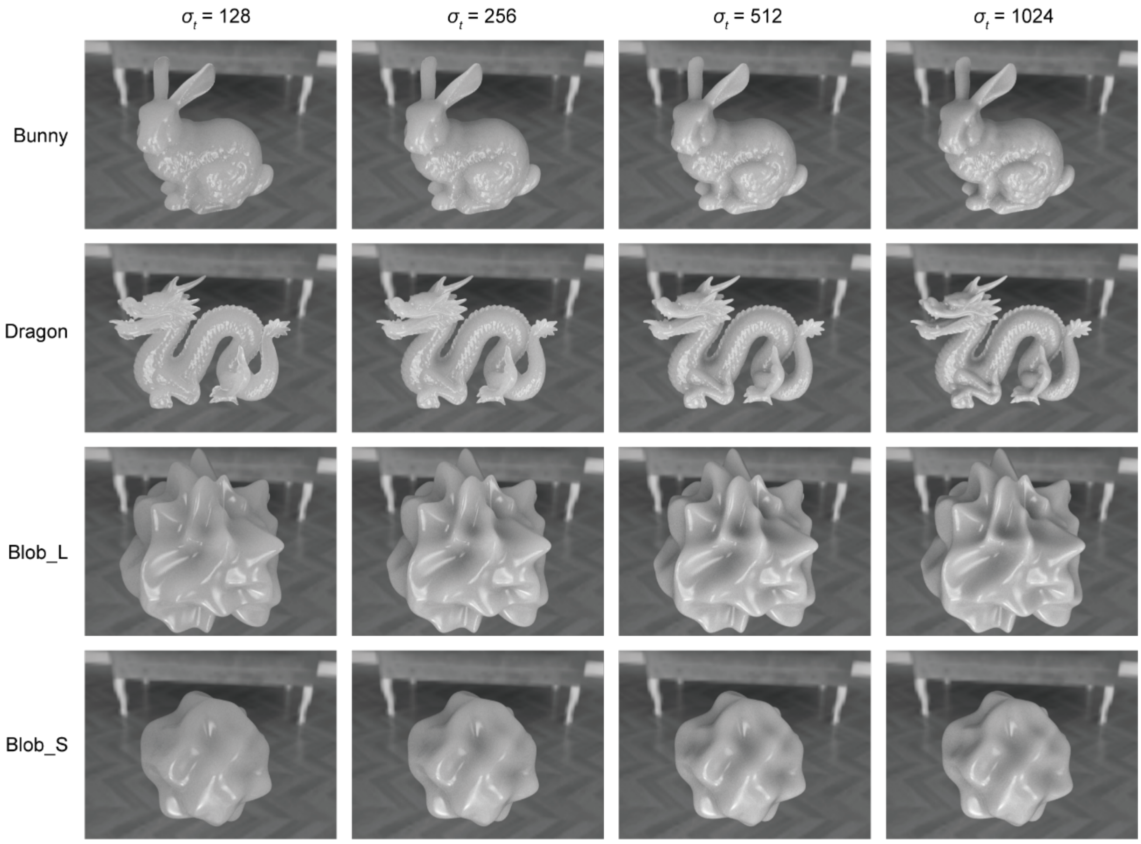

S1 Fig. Computer-graphics images under Specular condition.

Only single-angle images presented for the left eye are provided.
