# Supplementary material for: Top-down effects on translucency perception in relation to shape cues
Source: PLoS One. 2025 Feb 18;20(2):e0314439. doi: 10.1371/journal.pone.0314439 (PMC11835294; doi:10.1371/journal.pone.0314439)
Supplement: S3 Fig — (PDF) [file pone.0314439.s005.pdf]

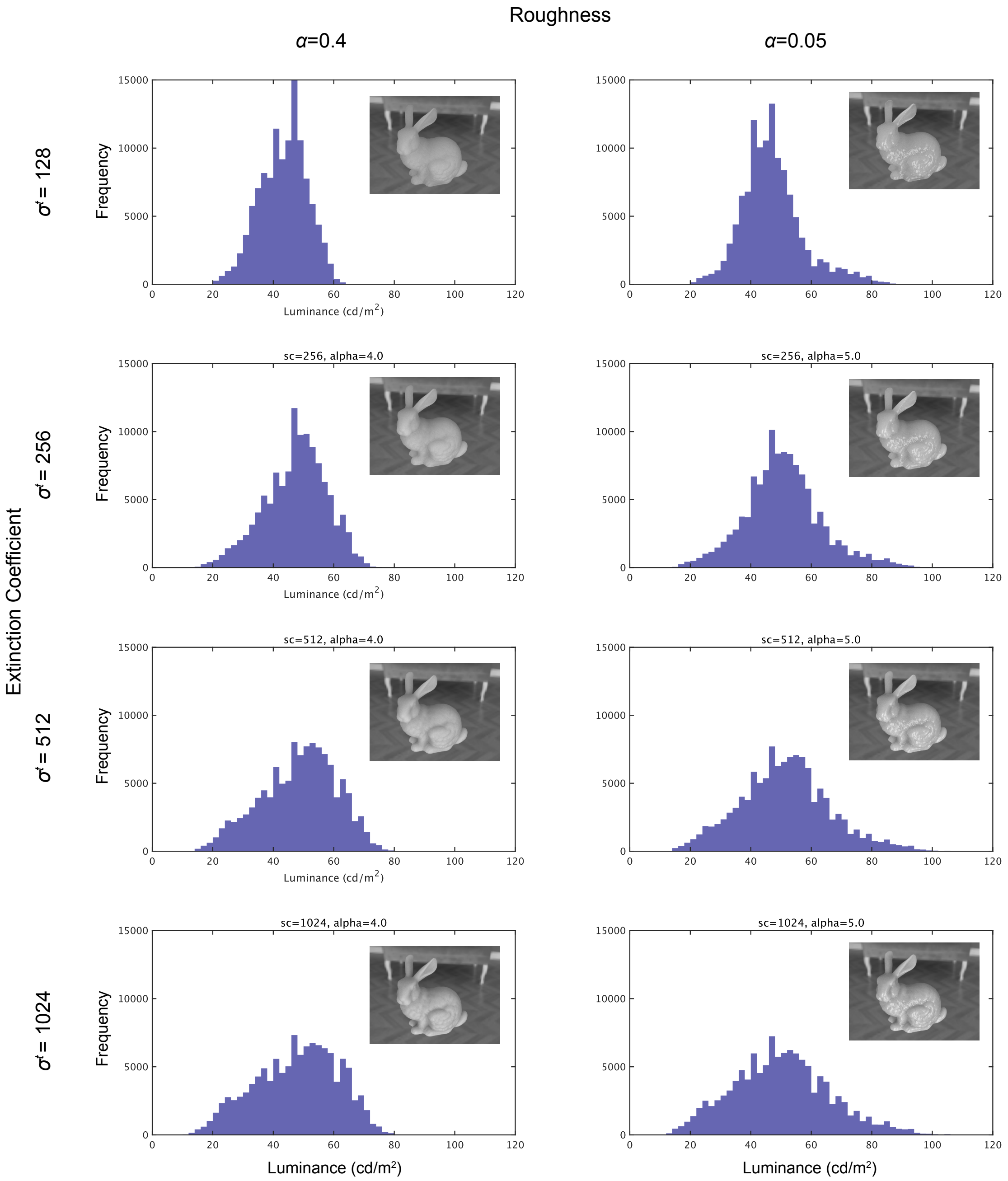

S3 Fig. Luminance histogram of rendered images.

The bunny images in S1 and S2 Figs are analyzed as examples. The analysis focuses exclusively on the object regions, excluding the background.
