# Supplementary material for: Top-down effects on translucency perception in relation to shape cues
Source: PLoS One. 2025 Feb 18;20(2):e0314439. doi: 10.1371/journal.pone.0314439 (PMC11835294; doi:10.1371/journal.pone.0314439)
Supplement: S4 Fig — (PDF) [file pone.0314439.s006.pdf]

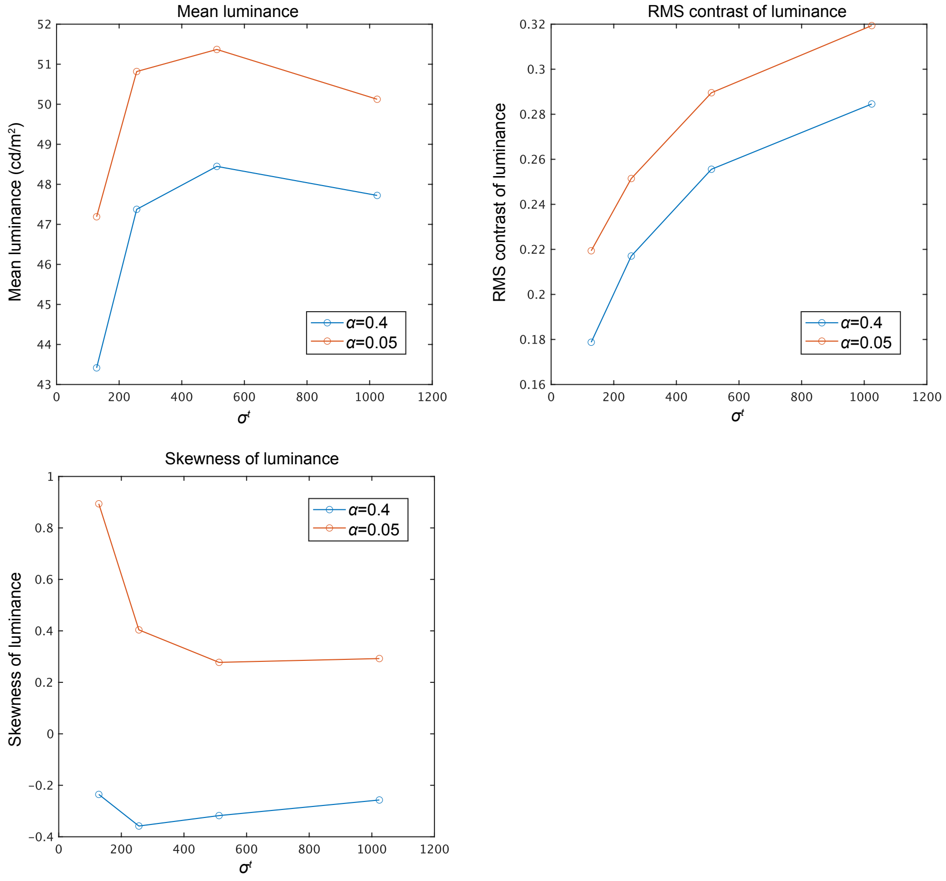


**S4 Fig. Basic luminance statistics of rendered images.**

These pixel-based statistics are derived from the luminance histograms shown in S3 Fig. RMS contrast is defined as the standard deviation of luminance divided by its mean.
